# Hepatoprotective Bioactivity of the Glycoprotein, Antrodan, Isolated from *Antrodia cinnamomea* Mycelia

**DOI:** 10.1371/journal.pone.0093191

**Published:** 2014-04-01

**Authors:** Yaw-Bee Ker, Chiung-Chi Peng, Wan-Lin Chang, Charng-Cherng Chyau, Robert Y. Peng

**Affiliations:** 1 Department of Applied Food Technology, Hungkuang University, Taichung, Taiwan, ROC; 2 Graduate Institute of Clinical Medicine, College of Medicine, Taipei Medical University, Taipei, Taiwan, ROC; 3 Research Institute of Biotechnology, Hungkuang University, Taichung, Taiwan, ROC; Taipei Medical University, Taiwan

## Abstract

Antrodan, a protein-bound polysaccharide isolated from *Antrodia cinnamomea* mycelia, was demonstrated to exhibit significant anti-inflammatory bioactivity *in vitro*. However, its role in hepatic injury *in vivo* still remains unclear. We hypothesized that antrodan may have beneficial hepatoprotective effects. To verify this, a lipopolysaccharide (LPS)-Sprague-Dawley rat model was used. Antrodan protected against liver damage by suppressing LPS-stimulated serum glutamine-oxaloacetic transaminase (GOT), glutamic-pyruvic transaminase (GPT), interleukin (IL)-6, hepatic thiobarbituric acid reactive substances (TBARS), nitric oxide (NO), inducible NO synthase (iNOS) and nuclear factor (NF)-κB, and by effectively alleviating the downregulated hepatic superoxide dismutase (SOD), catalase, and glutathione peroxidase (GSH-Px). Hematoxylin-eosin staining revealed that antrodan at a dosage of 40 mg/kg was able to alleviate LPS-induced liver damage to a normal status. In addition, we identified the partial main architectural backbone of antrodan to have a 1→3 linear β-glycosidic backbone of mannan linked by β-1→3 glucosidic branches. Conclusively, antrodan can potentially ameliorate liver damage *in vivo* by suppressing oxidative stress induced by LPS.

## Introduction

Beta-glucans are commonly found at high levels in fungi, yeast, oats, barley, and bacteria [1], [2]. Their potential bioactivities were reported to involve immunomodulation [3], [4]. anti-inflammation [5], anticarcinogenicity [6], [7], enhancement of natural killer cells [8], resistance against bacterial and parasitic infections [9], anti-diabetes, and reduction of associated cardiovascular risks [10]. Recently, the protein-bound polysaccharide K (trade name Krestin, or PSK) was approved in a phase 1 clinical trial of breast cancer by the US Food and Drug Administration in 2007 [11]. One of the important action mechanisms of PSK was described as being related to activation of antitumor immune responses [12]. Combining PSK with chemotherapy prolonged the survival rate of patients with gastric cancer, colorectal cancer, and small-cell lung carcinoma [13]. Generally terpenoids predominantly occur in *Antrodia cinnamomea* (syn. *A. camphorata*) (AC) fruiting bodies, in which 78 compounds have been identified [14]. AC fruiting bodies are effective against numerous diseases including hepatitis, diarrhea, abdominal pain, hypertension, and tumorigenic diseases [15]. To the present, the cited biological effects of AC are mostly limited to its triterpene content [15], [14], while the hepatoprotective bioactivity of AC polysaccharides has not been examined.

β-Glucans can act on several immune receptors including Dectin-1, complement receptor (CR)3 and Toll-like receptor (TLR)-2/6, and can also trigger groups of immune cells including macrophages, neutrophils, monocytes, natural killer (NK) cells, and dendritic cells and enhance phagocytosis [4]. The literature demonstrates that even a slight difference among glucans in terms of the glycosidic linkages, higher-order structure, molecular weight, solubility, protein and lipid pendants, and/or higher-order aggregates can result in great differences in innate immune activities [16], [17].

Decoking is thought to produce an extract comprising both triterpenes and a diversity of soluble polysaccharides, and we hypothesized that AC polysaccharides could contribute comparable, if not more-prominent, immunobioactivity that ultimately may be associated with certain promising hepatoprotective effects. To verify this, we carried out the present experiments.

## Materials and Methods

### Chemicals

Trypan blue, sodium nitrite (NaNO_2_), 3-(4,5-dimrthyl thiazol-2-yl)-2,5-diphenyl tetrazolium bromide (MTT), sodium bicarbonate, lipopolysaccharide (LPS, *Escherichia coli* 055:B5), 2′,7-dichlorofluoresein, and N-(1-naphthyl)-ethylenediamine dihydrochloride were procured from Sigma (St. Louis, MO, USA). Fetal bovine serum (FBS), an L-glutamine solution (100 mM), penicillin-streptomycin solution (5000 units/mL penicillin, 5 mg/mL streptomycin) were purchased from Biological Industries (Beit Haemek, Israel). Dulbecco's modified Eagle medium (DMEM) and a trypsin-EDTA solution were provided by Hyclone (Logan, UT, USA). A protein assay kit was a product of Bio-Rad (Hercules, CA, USA). Methanolic HCl (0.5 N), a Sylon HTP kit, and N,O-bis(trimethylsilyl)trifluoroacetamide (BSTFA) were products of Supelco (Bellefonate, PA, USA). The rat interleukin (IL)-6 enzyme-linked immunosorbent assay (ELISA) kit was supplied by R&D Systems (Minneapolis, MN, USA).

### Extraction and purification of antrodan and analysis of its glycosidic linkages

Antrodan, a kind of glycoprotein, was prepared from mycelia of *A. cinnamomea* according to our previous report [18]. In brief, defatted mycelial powder (1 kg) obtained by supercritical fluid extraction was refluxed with 20 L of double-distilled water (DDW) at 90°C for 2 h with constant stirring at 400 rpm. The extraction was repeated three times to deplete the water-soluble material. The residue was desiccated under *vaccuo* followed by extraction at a ratio of 1∶10 w/v with a hot alkaline solution (pH 9.0) at 80°C for 1 h. This extraction was repeated three times. After being cooled, the extracts were combined and filtered with the aid of aspiration. To the filtrate was added 1 N HCl to pH 4.0. A 2-fold volume of ethanol (95%) was then added. The precipitated polysaccharides were collected and re-purified in 4 L of hot water (90°C). In the final step, the soluble polysaccharides were re-precipitated by the addition of a 3-fold volume of ethanol (95%). The solution was left to stand for 4 h. The precipitate was collected, lyophilized, and pulverized (this was called AC-II). AC-II was further subjected to gel permeation chromatographic separation according to Carbonero et al. [19]. Briefly, 1 mL 0.05 N NaOH was added to AC-II (10 mg) and heated to 50°C while being stirred. After being cooled, the solution was centrifuged at 13,000×*g* for 5 min to eliminate any undissolved residue. The supernatant was subjected to a Sepharose CL-6B column (ℓ×id  = 82×3 cm) and eluted with DDW (adjusted to pH 11.0 with NaOH) at a flow rate 0.5 mL/min. The eluent was collected with a fractionator (Advantec SF-2120, Toyo Kaisha, Tokyo, Japan) at a velocity 1 tube/10 min. In total, 100 tubes were collected. The total sugar content of the eluent was determined, and the optical density (OD) at 280 nm was monitored. By referring to the OD at 280 nm, the main fractions (16^th^ to 40^th^) were selected and combined. The combined solution was lyophilized to yield a pure glucan, called “antrodan” in the text hereafter. To analyze the glycosidic linkage of the polysaccharide moiety (2 mg), partially methylated alditol acetates were prepared from permethyl derivatives by hydrolysis (with 2 M trifluoroacetic acid at 121°C for 2 h), reduction (with 10 mg NaBD_4_/mL for 2 h at room temperature), and acetylation (with acetic anhydride at 100°C for 1 h). A gas chromatographic-mass spectrometric (GC-MS) analysis was performed on an Agilent Gas Chromatograph 6890 (Santa Clara, CA, USA) connected to an Agilent 5973 Mass Selective Detector. Samples were dissolved in hexane and injected into the HP-5MS fused silica capillary column (ℓ×id  = 30 m×0.25 mm, ×25 μm, Agilent Technologies) at an injector temperature of 250°C. The helium carrier gas was operated at a flow rate of 1 ml/min. The oven, initially held at 100°C for 1 min, was programmed at a rate of 10°C/min to 200°C and held for 1 min at 200°C, and finally increased to 300°C at a rate of 15°C/min [20].

### Animal experiment

The animal experiment was approved by the Institutional Animal Care and Ethics Committee of Hungkuang University (Taichung, Taiwan). Thirty male Sprague-Dawley rats, aged 6 weeks and weighing 265∼287 g, were purchased from the BioLasCo Animal Center (Taipei, Taiwan). These rats were housed in stainless cages in an animal room maintained at 20±2°C, and a relative humidity of 65%±6%, with a 12/12-h light/dark cycle. These rats were fed ad libitum on basic granular chow (Fu-So Feed Stocks, Taichung, Taiwan) and water and acclimated in the animal room for the first week. The rats were randomly divided into five groups, with six in each group. The grouping involved the control, LPS control, antrodan control, LPS + low-dose antrodan (40 mg/kg) (antrodan L+LPS), and LPS + high-dose antrodan (80 mg/kg) (antrodan H+LPS) groups. Animals were separated and caged, with two or three rats in each cage. Antrodan was administered by gavage. The placebo group was fed basic chow only. Normal saline was used instead of antrodan in the LPS control. Access to chow and water was ad libitum. The entire experiment lasted 7 days. The initial and final body weights (BWs) were recorded. One day before being euthanized, LPS (5 mg/kg) (in saline) was intraperitoneally (i.p.) injected to induce acute hepatic injury. Then animals were anesthetized with CO_2_, and blood was collected from the hepatic portal vein and stored at 4°C. The heart, kidneys, and liver were excised, rinsed twice with phosphate-buffered saline (PBS), dewatered of soft skin tissues, weighed, and immediately immersed in liquid nitrogen for storage at −80°C for further use. The collected blood was centrifuged at 3000×*g* for 15 min to separate the serum, which was used in the following biochemical analyses.

### Analysis for serum glutamine-oxaloacetic transaminase (GOT) and glutamic-pyruvic transaminase (GPT)

Serum levels of GOT and GPT were assayed with Colorimetrics Slides (Fuji Dri-Chem Slide GOT/AST-PIII and Fuji Dri-Chem Slide GPT/ALT-PIII; Fuji, Japan) according to instructions given by the manufacturer.

### Analysis of serum IL-6 and nitrite/nitrate

Levels of serum IL-6 were determined by using a ELISA kit purchased from R&D Systems (Minneapolis, MN) according to the protocol provided by the manufacture. Briefly to a 96-well plate, 100 μL of capture antibody was added. The plate was tightly sealed and stored at room temperature (RT) overnight. After washes, the plates were blocked with Block Buffer at room temperature for 1 h. Authentic IL-6 or serum (100 μL) was added and left to react for 2 h at RT. Dilute detection antibody was loaded to each well, and the plate was incubated for an additional 2 h at RT. Avidin peroxidase (100 μL) was added and allowed to react for 30 min at ambient temperature. A substrate solution (ABTS liquid substrate solution) (100 μL) was added, and the mixture was allowed to react for 10 min while avoiding direct sunlight. The absorbance was read at 405 nm, with wavelength correction set at 650 nm using the ELISA reader (VersaMax, Molecular Devices, Sunnyvale, CA, USA). A calibration curve was similarly established using authentic IL-6. Serum nitrite (including nitrate) concentration was measured by a modified method of the Griess assay, described by Miranda et al. [21]. In brief, one hundred microliters of deproteinized serum samples were mixed with 100 μl of VCl_3_, rapidly followed by the addition of the Griess reagents (1% sulfanilamide dissolved in 5% phosphoric acid +0.1% naphthylethylenediamine dihydrochloride, 1∶1, v/v). The absorbance was immediately measured at 540 nm after the mixture being reacted for 15 min in dark at RT. Nitrite concentration was calculated using a NaNO_2_ standard curve and expressed as micromoles per liter.

### Protein extraction and quantification

The Bradford protein binding assay [22] was followed to determine the protein content in tissue. To 0.5 g of hepatic tissue, a 10-fold volume of phosphate-EDTA buffer (0.1 mM, pH 7.0) was added, homogenized for 20 min, and centrifuged at 3000×*g* for 15 min. The supernatant (1 mL) was transferred to a microcentrifuge tube and centrifuged at 4°C and 12,000×*g* for 10 min. The protein content of the supernatant was determined (called Hgn hereafter).

### Tissue superoxide dismutase (SOD) activity

The method of Marklund and Marklund [23] was followed to determine the tissue SOD activity. Briefly, to 40 μL of Hgn phosphate-EDTA buffer (50 mM, pH 7.0, 56 μL), a Triton X-100 solution (2%, pH 8.2, 96 μL) was added, mixed well, and centrifuged at 4°C and 12,000×*g* for 5 min. The supernatant was decanted and transferred to a 5-mL reaction vessel. Tris-HCl (3 mL, pH 8.2, 50 mM) was added and agitated for 5 min. To 10 μL of the mixture, methanolic pyrogallol (50 mM) was added, immediately mixed, and read at 325 nm. Readings were successively made every 15 s for a total period of 3 min. Water was used as the blank, and a similar protocol was conducted. The activity is expressed in U/mg protein. The following equation was used to calculate SOD activity:
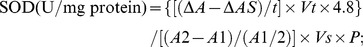
where ΔA is the change in the OD/min after adding water, ΔA_S_ is the change in the OD/min after adding the sample solution, V_t_ is the total volume of the reaction mixture (mL), 4.8 is the dilution extent, V_s_ is the total sample volume (mL), P is the total protein content (mg), A_1_ is the OD at 0 s after adding water, A_2_ is the OD at 120 s after adding water, and t is the time (min).

### Tissue catalase (CAT) activity

The method of Aebi [24] was adopted with slight modifications. Briefly, to 40 μL Hgn phosphate-EDTA buffer (50 mM, pH 7.0, 56 μL), a Triton X-100 solution (2%, pH 8.2, 96 μL) was added, mixed well, and centrifuged at 4°C and 12,000×*g* for 5 min. The supernatant (5 μL) was decanted and transferred to a 5-mL reaction vessel and diluted with phosphate-EDTA buffer (50 mM, pH 7.0, 5 mL). To the diluted solution (2 mL), H_2_O_2_ (1 mL, 30 mM) was added and immediately mixed, and the OD was read at 240 nm. Readings were successively taken every 15 s for a total reading period of 3 min. CAT activity is expressed as U/mg protein.

The following equation was used to calculate CAT activity:
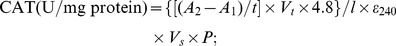
where A_2_ is the OD at 180 s, A_1_ is the OD at 0 s, t is the time (min), V_t_ is the total volume of the reaction mixture (mL), 4.8 is the dilution extent, ℓ is the light path (cm), ε_240_ is the extinction coefficient of 0.0395 mM^−1^cm^−1^, V_s_ is the total sample volume (mL), and P is the total protein content (mg).

### Tissue glutathione peroxidase (GSH-Px) activity

The method of Lawerence and Burk [25] was followed with slight modifications. Briefly, to 100 μL of Hgn, 800 μL potassium phosphate buffer (1 mM EDTA, 1 mM NaN_3_, 0.2 mM NADPH, 1 U/mL GSH reductase, and 1 mM GSH) was added and mixed well. The mixture was left at ambient temperature for 5 min to facilitate the reaction. To this reaction mixture, 100 μL of 2.5 mM H_2_O_2_ was added and mixed well. The OD was read at 340 nm. Readings were successively taken every 5 s for a period 3 min. The GSH-Px activity is expressed as U/mg protein.

The following equation was used to calculate GSH-Px activity:
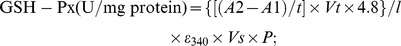
where A_2_ is the OD at 180 s, A_1_ is the OD at 0 s, t is the time (min), V_t_ is the total volume of the reaction mixture (mL), 4.8 is the extent of dilution, ℓ is the light path (cm), ε_340_ is the extinction coefficient of NADPH of 6.22 mM^−1^cm^−1^, V_s_ is the total sample volume (mL), and P is the total protein content (mg).

### Tissue thiobarbituric acid reactive substances (TBARS) level

The method of Buege and Aust [26] with slight modifications was used to determine the hepatic tissue TBARS level. Briefly, to 0.5 g of liver tissue, 5 mL of normal saline was added and homogenized. To determine the TBARS level, 2 mL of homogenate was transferred to a spiral tube, and successively 1.5 mL 0.01 N HCl and 0.5 mL 15% TCA +0.375% TBA +0.25 N HCl were added and agitated to mix it well. The mixture was heated to 100°C for 15 min, left to cool at ambient temperature, and centrifuged at 3000×*g* for 10 min. The supernatant was separated and read at 535 nm. A calibration curve was established using trimethylolpropane as the reference compound and treated similarly. The hepatic tissue TBARS level was calculated using this curve.

### Hematoxylin-eosin (H&E) staining

Liver tissue samples were collected at 24 h after LPS administration. After excision, the liver was cut into slices at a thickness of 0.5∼1.0 cm and immersed in a 10% neutral formalin solution for 3 days. Specimens were embedded in paraffin and frozen at 2∼8°C. The frozen slices were sliced with a rotary microtome to yield slices with a thickness of 4 μM. The microtomed slices were mounted onto microscopic glass slides, and treated with 50∼60°C water. After being tempered overnight in an oven held at 37°C, the slices were stained with H&E, sealed with fat-soluble gel, and examined microscopically (magnification, 200×).

### Western blot analysis of iNOS and NF-κB p65 in cytoplasm

Liver samples were homogenized and cytoplasmic proteins were isolated as following. One gram liver tissue was homogenized in 10 mL of ice-cold buffer A (10 mM HEPES, pH 7.9, 1.5 mM MgCl_2_, 10 mM KCl, 1 mM dithiothreitol, and 1 mM phenylmethylsulfonylfluoride) and incubated on ice for 10 min. The samples were then centrifuged at 850×*g* for 10 min at 4°C. The supernatants were discarded and the pellets were resuspended in 1 μL of buffer A with 0.1% Triton X-100 per μg of tissue, incubated for 10 min on ice and centrifuged as described above. The supernatant was removed and saved as the cytoplasmic fraction. The cytoplasmic protein was used for detection of inducible nitric oxide synthase (iNOS) and NF-κB p65 in Western blot analysis. Sample (30 μg of protein) was subjected to SDS-polyacrylamide gel electrophoresis (8% gel for iNOS and 12% for NF-κB p65). Proteins in the gel were transferred to a polyvinylidene difluoride membrane by electroblotting, and the membrane was incubated sequentially with primary antibodies that recognize iNOS (BD Biosciences, San Diego, CA; 1∶2000), NF-κB p65 (Millipore, Billerica, MA; 1∶500) and β-actin (Cell Signaling Technology, Danvers, MA; 1∶5000) for 2 h, and horseradish peroxidase-conjugated secondary antibody for 1 h at room temperature. After washing with phosphate-buffered saline (PBST), goat anti-rabbit IgG horseradish peroxidase (HRP)-conjugated secondary antibodies (Santa Cruz) (1∶1000 dilution in PBST) were incubated for1 h at roomtemperature. Immunoreactive proteins were detected using an enhanced chemiluminescence kit (ECL, PerkinElmer, Waltham, MA), and the relative expression of the protein bands was quantified by densitometry with ImageQuant TL software (GE Healthcare).

### Statistical analysis

Data obtained were statistically treated with a one-way analysis of variance (ANOVA). Tukey's test or a least significant different (LSD) test was used to analyze differences in significance. *P*<0.05 was considered to indicate a significant difference between groups.

## Results

### Characteristics of antrodan

**Figure 1:**
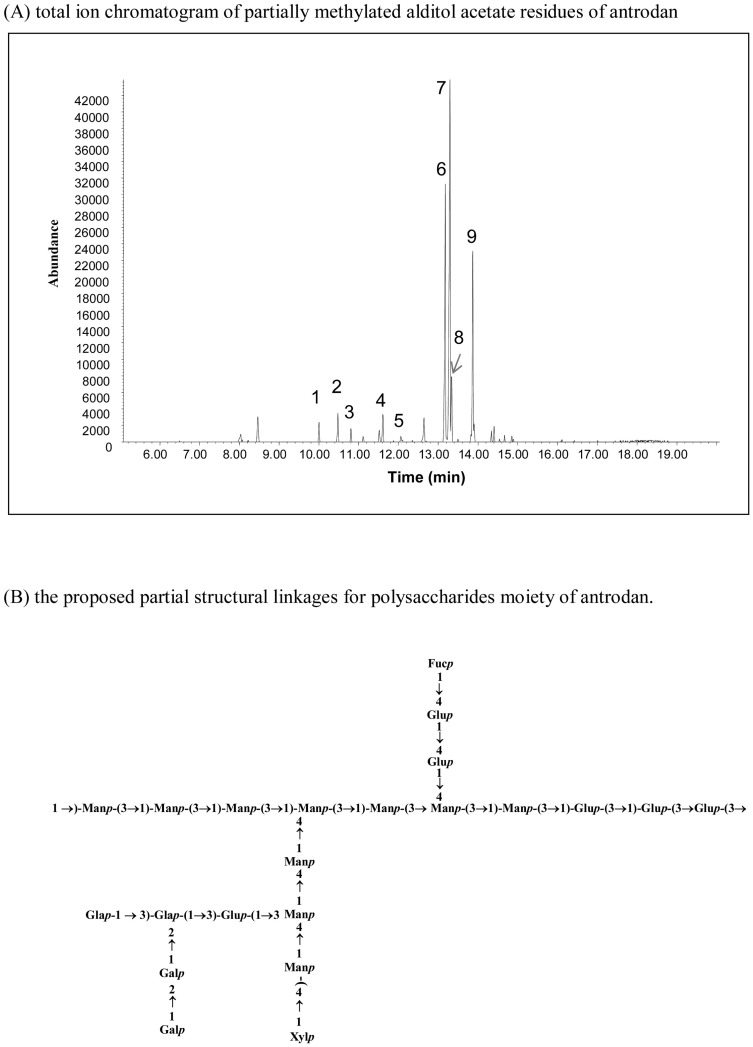
Total ion chromatogram of partially methylated alditol acetate residues of antrodan by a GC/MS analysis (A) and the proposed partial structural linkages of the polysaccharide moieties of antrodan (B).

The original yield of the crude AC-II fraction was 12.0%±0.3%, and the yield of the purified product was 9.2%±0.4%, which is hereafter called “antrodan”. The yield of antrodan was slightly lower than that previously reported [18]. Recently, we identified antrodan to be a glycoprotein with a molecular weight of 442±11 kDa which contains 14.10% carbohydrates, 71.0% protein, and a profound content of uronic acid (152.6±0.8 mg/g) [18]. In order to determine the nature of the linkages among different monosaccharides in the polysaccharide moiety of antrodan, the reduced polymers were permethylated and subjected to a GC-MS analysis (Fig. 1A). In the analysis of sugar linkages, the compound 2,4,6-Me_3_-Man*p* was found to be the most abundant among all monosaccharide derivatives. The second most abundant was 2,4,6-Me_3_-Glc*p* (Table 1). From the overall relative amounts of these derived monosaccharides, we suggest that antrodan exhibited mainly an architecture of a mannose backbone connected to glucose branches by 1,3-glycosidic linkages (Fig. 1B).

**Table 1:** Analysis for the glycosidic linkages present in the polysaccharide moieties of antrodan.

Peak No.^1^	Methylated sugars	Mass fragmentation (*m/z*)^2^	Glycosidic linkage	Peak area (%)
1	2,3,4-Me_3_-Glc	43, 118, 129, 102, 87, 71, 161	→6)-Glc-(1→	2
2	2,3,4-Me_3_-Xyl	43, 101, 102, 117, 118, 88, 73, 161	T-Xyl-(1→	4
3	2,3,4-Me_3_-Fuc	43, 118, 115, 102, 89, 131, 101, 175	T-Fuc-(1→	2
4	2,3,6-Me_3_-Glc	43, 118, 129, 117, 87, 130, 88, 102	→4)-Glc-(1→	4
5	2,3,4,6-Me_3_-Gal	43, 102, 45, 118, 129, 145, 161, 162	T-Gal-(1→	1
6	2,4,6-Me_3_-Glc	43, 118, 129, 101, 161, 234, 87	→3)-Glc-(1→	26
7	2,4,6-Me_3_-Man	43, 118, 113, 233, 102, 99, 87	→3)-Man-(1→	45
8	2,3,6-Me_3_-Man	43, 118, 129, 101, 161, 234, 74, 87	→4)-Man-(1→	5
9	4,6-Me_2_-Gal	43, 118, 129, 87, 143, 59, 74, 185, 232	2,3)-Gal-(1→	11

1 Peak numbers correspond to Fig. 1A.

2 Mass fragmentation obtained from the GC-MS analysis, after methylation, total acidic hydrolysis, reduction with NaBD_4_, and acetylation. Ion fragments are presented in the order of high to low abundances.

### Variations of BWs and LWs and the LW/BW ratio

After 7 days of feeding, the apparent BWs did not significantly vary among all rat groups (Table 2). The absolute LW was found to have been highly increased in the antrodan L+LPS group, but was substantially suppressed in the antrodan H+LPS group to a level comparable to that of the antrodan control (Table 2). However, the LW/BW ratio was found to have been significantly reduced by LPS treatment. Antrodan alone or the antrodan+LPS combination revealed no effect on the LW/BW ratio (Table 2).

**Table 2:** Effects of antrodan on various body and organ weights.^1^^,^ 2

Parameter	Control	LPS	Antrodan	Antrodan L+ LPS	Antrodan H + LPS
**Body weight (g)**					
**Initial**	260.50±8.35^a^	263.40±7.60^a^	260.25±10.37^a^	262.6±13.48^a^	260.0±12.39^a^
**Final**	310.12±6.76^a^	314.25±9.00^a^	308.25±17.27^a^	305.0±20.68^a^	304.5±20.4^a^
**Liver weight (g)**	10.75±1.65^b^	10.83±1.42^b^	11.15±1.43^b^	11.70±0.80^a^	11.32±1.13^b^
**Liver to body weight (%)**	3.75±0.54^ab^	3.44±0.43^b^	3.69±0.29^ab^	3.85±0.33^a^	3.73±0.45^a^

1 LPS, 5 mg/kg lipopolysaccharide; Antrodan, antrodan control at 40 mg/kg; Antrodan L, 40 mg/kg + LPS; Antrodan H, 80 mg/kg + LPS.

2 Values are expressed as mean ± S.D. (*n* = 6). Within the same row, different superscripts indicate significant differences between treatments (*p*<0.05) by using one way ANOVA followed by the post-hoc LSD test.

### Antrodan suppressed the hepatic tissues LPS-induced elevation of GOT, GPT, IL-6, and TBARS

**Figure 2:**
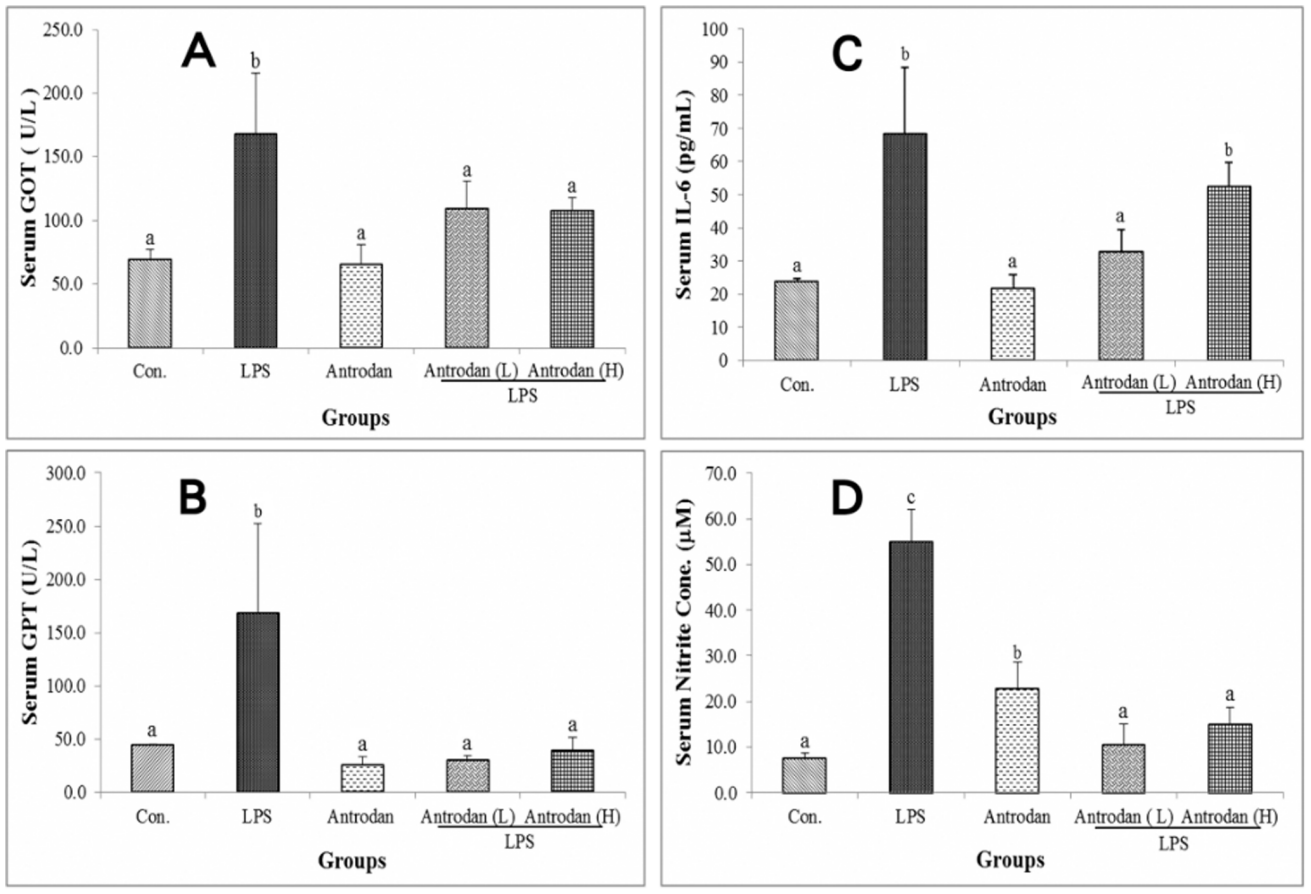
Effect of antrodan on lipopolysaccharide (LPS)-induced serum glutamine-oxaloacetic transaminase (GOT) (A), glutamic-pyruvic transaminase (GPT) (B), interleukin (IL)-6 (C) and nitric oxide (NO) (D) levels. Con, control; LPS, 5 mg/kg LPS; Antrodan, 40 mg/kg antrodan; Antrodan L+LPS, 40 mg/kg antrodan + LPS; Antrodan H+LPS: 80 mg/kg antrodan + LPS. Values are expressed as the mean ± S.D. (*n* = 6). One way ANOVA is followed by the post-hoc LSD test. Different letters indicate a significant difference (*p*<0.05).

LPS highly stimulated serum GOT, GPT, and IL-6 levels to 168±45 U/L, 160±76 U/L, and 68.3±20.2 pg/mL, respectively (Fig. 2A–2C). Antrodan at a dose of 40 mg/kg almost effectively alleviated these adverse effects (Fig. 1A–1C) (*p*<0.05)). Interestingly, as to inhibition of IL-6 production, antrodan alone showed a rather prominent suppressive effect. However, in treated groups, the higher dose of antrodan was shown to be much inferior in a dose-dependent manner (Fig. 2C) (*p*<0.05).

### Antrodan alleviated the *in vivo* LPS-induced suppression of hepatic antioxidant enzymes activities

**Figure 3:**
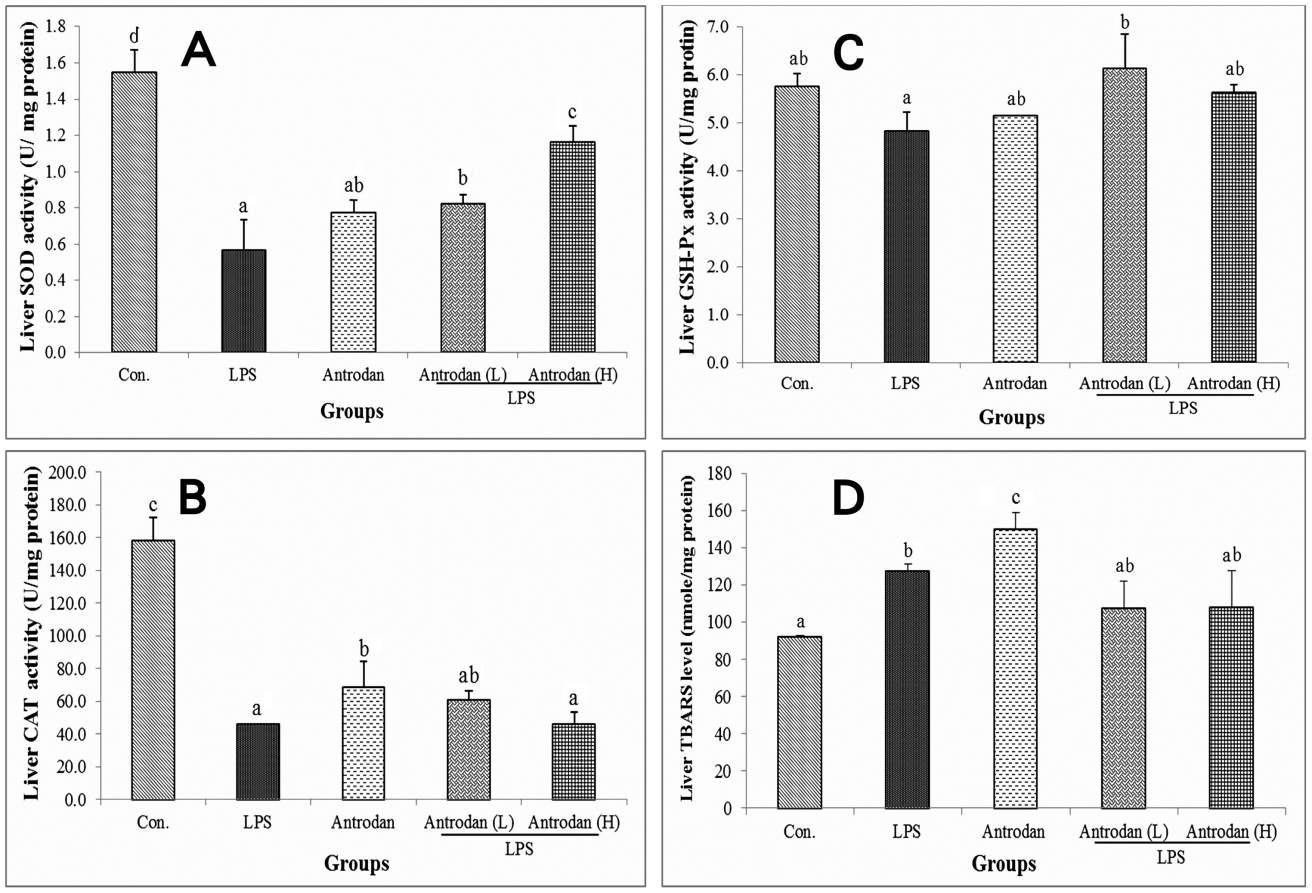
Effect of antrodan on the lipopolysaccharide (LPS)-induced hepatic antioxidative capability. (A) Superoxide dismutase, (B) catalase, (C) glutathione peroxidase (GSH-Px), and (D) thiobarbituric acid reactive substances (TBARS). Con, control; LPS, 5 mg/kg LPS; Antrodan, 40 mg/kg antrodan; Antrodan L+LPS, 40 mg/kg antrodan + LPS; Antrodan H+LPS, 80 mg/kg antrodan + LPS. Values are expressed as the mean ± S.D. (*n* = 6). One way ANOVA is followed by the post-hoc LSD test. Different letters indicate a significant difference (*p*<0.05).

LPS treatment suppressed the hepatic SOD level to 0.56±0.17 U/mg protein after 24 h. In rats treated with antrodan alone, the level was 0.74±0.06 U/mg protein. Thus, compared to control (1.54±0.12 U/mg protein), LPS reduced SOD by about 77% (*p*<0.05) and antrodan by 52% (*p*<0.05) (Fig. 3A). The low dose of antrodan did not have any improving effect, while the higher dose apparently showed a higher recovery efficiency (Fig. 2A).

After treatment for 24 h, LPS reduced the CAT activity by 71% compared to the control (46.17±0.24 vs. 158.26±14.29 U/mg protein). Antrodan alone suppressed the CAT level to 68.54±15.74 U/mg protein, a 57% decrease (*p*<0.05). It is worth noting that antrodan L+LPS and antrodan H+LPS showed no better effect, and respective CAT activities were still 61.55±4.68 and 46.23±7.18 U/mg protein (Fig. 3B). GSH-Px activity was not affected by either LPS or antrodan. GSH-Px activities were 5.76±0.26, 4.83±0.4, 5.15±0.03, 6.13±0.72, and 5.64±0.15 U/mg protein in the control, LPS control, antrodan control, antrodan L+LPS, and antrodan H+LPS groups, respectively. LPS inhibited hepatic GSH-Px. Antrodan alone seemed to have little effect on rescuing hepatic GSH-Px. However in counteracting LPS-induced GSH-Px inhibition, the lower dose of antrodan (40 mg/mL) seemed to be more effective than the higher dose (Fig. 3C).

### Antrodan alleviated the *in vivo* LPS-induced suppression of hepatic TBARS

LPS significantly stimulated hepatic TBARS to 127.16±4.10 nmole/mg protein compared to 92.11±0.70 nmole/mg protein in the control, yielding an increase of 38% (*p*<0.05) (Fig. 3D). To our surprise, antrodan alone further raised the hepatic TBARS to 150.14±8.64 nmole/mg protein for a 63% increase (*p*<0.05). In contrast, in the antrodan L+LPS and antrodan H+LPS groups, TBARS levels were significantly ameliorated to 107.48±14.37 and 107.95±19.84 nmole/mg protein, respectively. It seemed that a low dose of antrodan (40 mg/kg) had a full-strength alleviating effect (Fig. 3D).

### Antrodan alleviated LPS-induced suppression of serum NO *in vivo*

LPS highly stimulated serum NO to 55.5±6.0 μM (Fig. 3E). Antrodan moderately alleviated this adverse effect at a dose of as low as 40 mg/kg and completely alleviated the effect at a dose of 80 mg/kg in the combination treatments of antrodan L+LPS and antrodan H+LPS (Fig. 3E) (*p*<0.05).

### H&E staining of hepatic tissues

**Figure 4:**
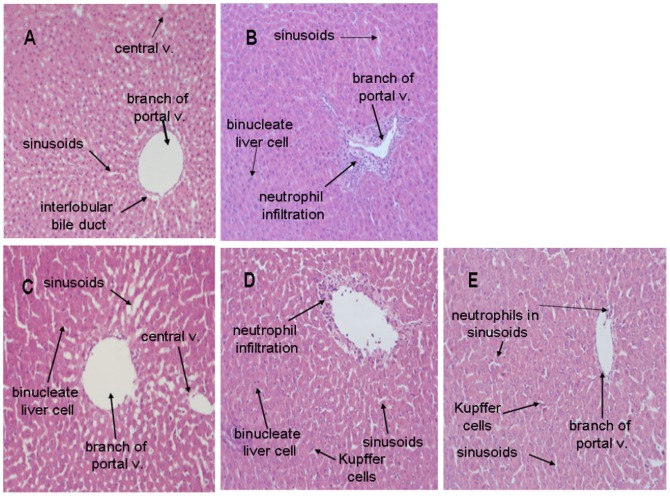
H&E staining of liver tissues. (A) Control; (B) tissue treated with 5 mg/kg (i.p.) lipopolysaccharide (LPS) showing severe inflammatory cell infiltration and markedly diffuse cellular infiltration, and vacuolar degeneration in some hepatocytes; (C) treated with 40 mg/kg antrodan; (D) pretreated with 40 mg/kg antrodan by gavage and then with LPS, showing focal areas of liver parenchyma with neutrophil infiltration and necrosis; (E) animal pretreated with 80 mg/kg antrodan by gavage and then with LPS, showing no pathological changes except for a few neutrophils in the sinusoids. Antrodan at 40 mg/kg was revealed to be hepatoprotective against the inflammatory abuse by LPS. (magnification, all 200×).

The histopathological examination revealed no morphological alterations in either the control (Fig. 4A) or antrodan group (Fig. 4C). In contrast, LPS damaged hepatic tissues and increased neutrophils and lymphocytes, inducing hepatic necrosis and cell lysis, releasing a tremendous number of nuclei and eliciting degenerative death of hepatic cells (Fig. 4B) (indicated by arrows). Oral administration of antrodan daily for 7 days significantly improved the state of hepatic necrosis while the antrodan control group shown as non-toxic injury on liver (Fig. 4D–E). Antrodan was found to be dose-responsively beneficial in alleviating cytotoxicity. At the lower dose, antrodan-treated liver leucocytes aggregated around blood vessels, and leucocyte infiltration was found in hepatic cells (Fig. 4D). However, the phenomenon was much highly improved over that found in the LPS-treated group. Interestingly, the high dose of antrodan aggravated LPS-induced hepatic damage. Inflammation with neutrophil aggregation was very common (indicated by arrows in Fig. 4E).

### Western blot of cytosolic NF-κB and iNOS

**Figure 5:**
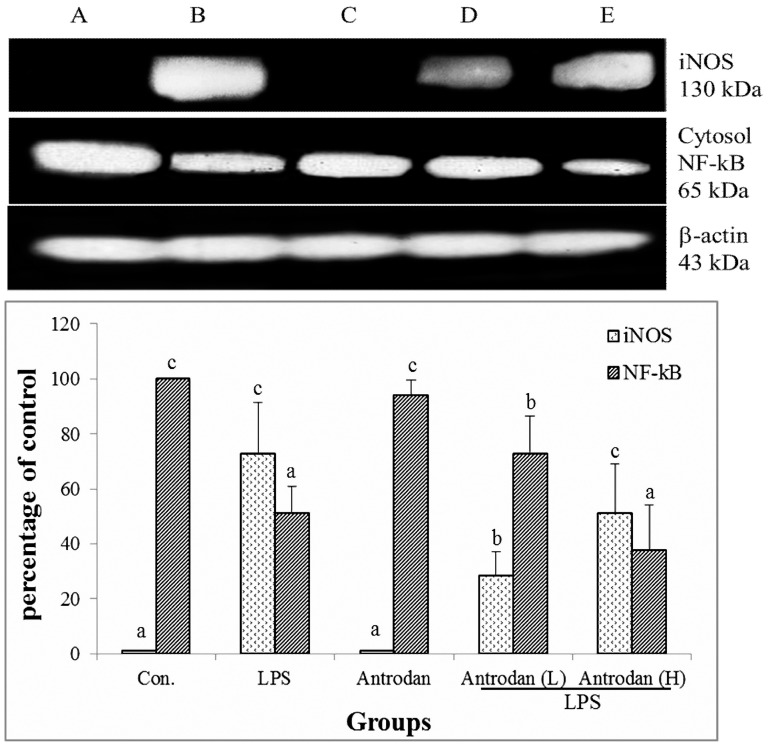
Effect of antrodan on the activation of cytoplasmic NF-κB (p65) protein and the expression of iNOS in lipopolysaccharide (LPS)-stimulated rat liver tissue by Western blot analysis. Con, control; LPS, 5 mg/kg LPS; Antrodan, 40 mg/kg antrodan; Antrodan L+LPS, 40 mg/kg antrodan + LPS; Antrodan H+LPS, 80 mg/kg antrodan + LPS. Values are expressed as the mean ± S.D. (*n* = 6). One way ANOVA is followed by the post-hoc LSD test. Different letters indicate a significant difference (*p*<0.05).

LPS highly upregulated cytosolic NF-κB and substantially downregulated iNOS activity (Fig. 4). Antrodan alone showed comparable levels of NF-κB and iNOS as the control. Both antrodan L+LPS and antrodan H+LPS moderately suppressed iNOS upregulation (Fig. 5). Amazingly, the higher dose of antrodan was found to be less effective than treatment with the lower dose (Fig. 5).

## Discussion

Recently, we showed antrodan characteristically to be a protein-bound polysaccharide in which β-glucans occupied a higher percentage (14.20%) than α-glucans (1.45%). In addition, a large amount of uronic acid (152.6 mg/g) coexists in the complex [18]. The high content of uronic acid may contribute to antrodan's bioactivities. A high uronic acid content in polysaccharide conjugates was shown to exhibit stronger reactive oxygen species-scavenging activities [27]. Certain polysaccharide-protein complexes obtained from mushrooms are capable of stimulating the non-specific immune system, exerting antitumor activities through stimulating a host's defense mechanism [28]. A similar structural type glucoxylan-protein complex prepared from *Hericium erinaceus* was been found to have immunomodulatory activity [29].

Nonetheless, the β-glucan content of antrodan is slightly lower compared to yeast which has a β-(1→3) glucan content of 45.47% [18]. Importantly, the animal experiment revealed that antrodan was nontoxic and safe in nature, hence feasible for use in nutraceutical therapy (Table 2). Liu et al. [30] demonstrated that proteins in polysaccharides directly contribute to free radical-scavenging activities. Protein-bound polysaccharides extracted from fruiting bodies of *Ganoderma atrum* also revealed multiple strong antioxidant activities [31]. As mentioned, we showed antrodan to be a glycoprotein [18]. Now we found that linkages of the polysaccharide moieties in antrodan exhibited mostly backbones of 1→3 linear β-glycosidic chains of mannose linked by β-1→3 glucose branches (Table 1, Fig. 1B). These might contribute to certain specific biological functions of antrodan. Animal studies demonstrated that the 1→3 linear β-glycosidic backbone of β-glucans in fact cannot be readily digested *in vivo* [4]. Most β-glucans enter the proximal small intestine, and some are captured by macrophages. First, they are internalized and fragmented within cells and then transported by macrophages to the marrow and endothelial reticular system. There the reduced β-glucan fragments are eventually released by macrophages and taken up by other immune cells leading to a variety of immune responses [4].

IL-6 has been found both in local and systemic acute inflammatory responses. However, the main sources of IL-6 *in vivo* are stimulated monocytes/macrophages, fibroblasts, and vascular endothelial cells [32]. In liver tissues, different sources of IL-6 present different functions. The IL-6 in hepatocytes induces STAT3 activation and plays important roles in hepatoprotection and liver regeneration. In Kupffer cells, IL-6 induces transient STAT3 activation and contributes to the pro-inflammatory response. In sinusoidal endothelial cells or hepatic stellate cells, IL-6 induces STAT3 activation and subsequently promotes cell survival [33]. Therefore, IL-6 could have anti and pro-inflammatory functions and the circulating levels of IL-6 are directly and closely correlated with the severity of disease [34]. IL-6 in serum was determined in this study would be more corresponding to reality of LPS-induced inflammatory responses of rats. Direct contact of β-glucans present in fungal cell walls is sufficient to trigger the rapid synthesis and secretion of the IL-6 protein, a post-transcriptional regulator of IL-6 in response to fungal extracts mediated by the p38 mitogen-activated protein kinase pathway [35]. β-Glucans are also beneficial against diabetes and associated cardiovascular risks [10].

LPS exerts oxidative stress and damages rat livers [35]. Activities of alanine aminotransferase (ALT or GPT), aspartate aminotransferase (AST or GOT), alkaline phosphatase (ALP), lactate dehydrogenase (LDH), and levels of serum total bilirubin, total protein, TNF-α, and IL-1β all increased [34]. Consistently, we showed that LPS stimulated activities of serum GOT, GPT, IL-6, and NO (Fig. 2A-D). Antrodan was found to be beneficial to the LPS-damaged liver (Table 2) as evidenced by suppressed serum levels of GOT and GPT (Figs. 1B, 2A).

β-Glucan stimulates lymphocyte formation in the injured body, producing the cytokine, IL-1, and an immunoglobulin M (IgM) antibody and adjusting the immune function [4]. Hepatic levels of TBARS, protein carbonyl content (PCC), CAT, glutathione peroxidase (GSH-Px), and myeloperoxidase (MPO) were stimulated by LPS. Conversely, reduced glutathione (GSH) and SOD were suppressed by LPS [36]. Yokoyama et al. [37] demonstrated that LPS administration increased the conversion of oxidized cytochrome c into reduced cytochrome c in the perfusate, indicating that superoxide anions had formed in the hepatic sinusoids, suggesting that superoxide anions in hepatic sinusoids may be one of the pathogenic factors behind damage to epithelial cells of hepatic sinusoids caused by LPS [37]. LPS administration induced lipoperoxidation and depletion of antioxidant enzyme activities such as SOD, CAT, and GSH-Px activities [38]. We found that LPS downregulated hepatic SOD, CAT, and GSH-Px (Fig. 3A–C), resulting in substantial elevation of the hepatic TBARS level (Fig. 2D). Astonishingly, although the higher dose (80 mg/kg) of antrodan showed a better effect for alleviating hepatic SOD (Fig. 3A), it was less effective for serum IL-6 (Fig. 1C), hepatic CAT (Fig. 3B), and serum NO (Fig. 3E). In contrast, hepatic GSH-Px was almost completely ameliorated by antrodan (Fig. 3C).

One current hypothesis for the molecular mechanisms of septic shock is that enhanced NO production by mitochondrial (mt) NOS leads to excessive peroxynitrite (ONOO^-^) production and protein nitration in the mitochondrial matrix, resulting in mitochondrial dysfunction and contractile failure [39].

Antrodan showed a promising *in vivo* suppressive effect on LPS-induced production of serum NO. We speculated that the potent biological effect by antrodan of scavenging NO might be attributed to its high uronic acid content (Fig. 3E, Table 1). LPS increased (*p*<0.05) TBARS, 13,14-dihydro-15-keto-prostaglandin F(2α) and NO [40]. Surface chemiluminescence was suggested to be a useful assay to assess inflammation and oxidative stress *in situ* in the liver and skeletal muscles. Liver chemiluminescence in inflammatory processes and phagocyte chemiluminescence were found to spectrally differ from spontaneous liver chemiluminescence with increased emission at 440∼600 nm [39]. Boveris et al. pointed out the possibility that NO^−^ and ONOO^−^ could participate in reactions leading to the formation of excited species [39].

While evidence for ‘baseline’ iNOS expression has been elusive, IRF1 and NF-κB-dependent activation of the iNOS promoter supports inflammation-mediated stimulation of this transcript. iNOS produces large quantities of NO upon stimulation, such as by proinflammatory cytokines (e.g., IL-1, TNF-αα, and interferon-γ) [41]. Induction of high-output iNOS usually occurs in an oxidative environment, and thus high levels of NO have the opportunity to react with superoxide leading to peroxynitrite formation and cell toxicity. These properties may define the roles of iNOS in host immunity, enabling its participation in antimicrobial and antitumor activities as part of oxidative bursts of macrophages [42].

The phenomena that antrodan alone actively raised the hepatic TBARS to a high level but was only moderately effective in counteracting LPS-induced TBARS elevation (Fig. 3D) imply dual bioactivities of antrodan to simultaneously act as both an oxidative stress-defensive (Fig. 3A, 3B) and oxidative stress-inducing agent (Fig. 2C, D). LPS induced hepatotoxicity in rats with subacute pretreatment, and part of its action mechanism was associated with iron sequestration from the plasma to the liver compartment [38]. H&E staining indicated that antrodan enhanced hepatic cell proliferation (Fig. 3C). Antrodan alleviated these cytotoxicities (Fig. 4A–D). Alternatively, the cause of serum NO upregulation was due to upregulation of iNOS, and the liver damage exerted by LPS was attributed to the upregulated iNOS and downregulated cytosolic NF-κB (Fig. 4). Antrodan moderately ameliorated these expressions (Fig. 5). Similar to the above-mentioned findings, a high dose of antrodan conversely aggravated LPS-induced hepatic damage where inflammation with neutrophil aggregation was very common (indicated by arrows in Fig. 4).

It is worth noting that the active polysaccharide fractionated from the fruiting body of *Agaricus blazei* exhibited main components of FI0, a β-(1→6)-; (1→3)-β-D-glucan; FA-1, an-α acidic (1→6)-; (1→4)-α-D-glucan; FA-1, an acidic β-(1→6)-; α-(1→3)-D-glucan; and FA-2, a β-acidic RNA-protein complex [43]. The purified fraction, HM3-G (MW 380 kDa) was shown to be associated with the highest tumoricidal activity. Its main sugar is glucose (90%). Structurally, its main component is (1→4)-alpha-D-glucan with (1→6)-beta branching, in a ratio of approximately 4∶1 [43]. Extensive investigation of cell-surface carbohydrates in tumor cells revealed two opposing roles in tumor metastasis: depending on their structures: carbohydrates can either promote or suppress metastasis. N-Glycans (N-acetylglucosamine attached to asparagine) were shown to play several important roles in tumor metastasis [44]–[46]. On the other hand, Core2 O-glycans allow tumor cells to evade NK cells of the immune system and survive longer in the circulatory system, thereby promoting tumor metastasis. Core3 O-glycans or O-mannosyl glycans suppress tumor formation and metastasis by modulating integrin-mediated signaling [47].

In conclusion, a low dose of antrodan (40 mg/kg) can act as a promising hepatoprotective agent. In addition, we identified the partial main architectural backbone of antrodan to have a 1→3 linear β-glycosidic backbone of mannan linked by β-1→3 glucosan branches. The findings of certain adverse effects that might be caused by the higher dose of antrodan inspired us to conduct further studies on i) reducing the molecular weight (or molecular size); ii) eliminating most of the Core2 O-glycans, if any; and iii) eliminating the N-glycan content, if any. However, according to previous experiences [4], the strategy of reducing the molecular weight might be feasible to obtain a more potential candidate in treating LPS-induced inflammatory disease.

## References

[1] Chen J, Seviour R (2007) Medicinal importance of fungal beta-(1→3), (1→6)-glucans. Mycol Res 111: 635–652. 17590323 10.1016/j.mycres.2007.02.011

[2] Bohn JA, BeMiller JN (1995) (1→3)-β-D-glucans as biological response modifiers: a review of structure-functional activity relationships. Carbohydr Polym 28: 3–14.

[3] Tsukagoshi S, Hashimoto Y, Fujii G, Kobayashi H, Nomoto K, et al (1984) Krestin (PSK). Cancer Treat Rev 11: 31–55. 10.1016/0305-7372(84)90005-7 6238674

[4] Chan GCF, Chan WK, Sze DMY (2009) The effects of β-glucan on human immune and cancer cells (review). J Hematol Oncol 2: 25. 19515245 10.1186/1756-8722-2-25 PMC2704234

[5] Jedinak A, Dudhgaonkar S, Wu QL, Simon J, Sliva D (2011) Anti-inflammatory activity of edible oyster mushroom is mediated through the inhibition of NFκB and AP-1 signaling. Nutri J 10: 52. 10.1186/1475-2891-10-52 PMC3120742 21575254

[6] Song KS, Li G, Kim JS, Jing K, Kim TD, et al (2011) Protein-bound polysaccharide from *Phellinus linteus* inhibits tumor growth, invasion, and angiogenesis and alters Wnt/b-catenin in SW480 human colon cancer cells. BMC Cancer 11: 307. 21781302 10.1186/1471-2407-11-307 PMC3154178

[7] Hong F, Yan J, Baran JT, Allendorf DJ, Hansen RD, et al (2004) Mechanism by which orally administered (beta)-1→3-glucans enhance the tumoricidal activity of antitumor monoclonal antibodies in murine tumor models. J Immunol 173: 797–806. 15240666 10.4049/jimmunol.173.2.797

[8] Takimoto H, Wakita D, Kawaguchi K, Kumazawa Y (2004) Potentiation of cytotoxic activity in naive and tumor-bearing mice by oral administration of hot-water extracts from *Agaricus blazei* fruiting bodies. Biol Pharm Bull 27: 404–406. 14993810 10.1248/bpb.27.404

[9] Yun CH, Estrada A, Van KA, Park BC, Laarveld B (2003) Beta-glucan, extracted from oat, enhances disease resistance against bacterial and parasitic infections. FEMS Immunol Med Microbiol 35: 67–75. 12589959 10.1016/S0928-8244(02)00460-1

[10] Chen J, Raymond K (2008) Beta-glucans in the treatment of diabetes and associated cardiovascular risks. Vasc Health Risk Manag 4: 1265–1272. 19337540 10.2147/vhrm.s3803 PMC2663451

[11] Torkelson CJ, Sweet E, Martzen MR, Sasagawa M, Wenner CA, et al. (2012) Phase 1 clinical trial of *Trametes versicolor* in women with breast cancer. ISRN Oncol doi:10.5402/2012/251632 10.5402/2012/251632 PMC3369477 22701186

[12] Akagi J, Baba H (2010) PSK may suppress CD57^+^ T cells to improve survival of advanced gastric cancer patients. Int J Clin Oncol 15: 145–152. 20229169 10.1007/s10147-010-0033-1

[13] Maehara Y, Tsujitani S, Saeki H, Oki E, Yoshinaga K, et al (2012) Biological mechanism and clinical effect of protein-bound polysaccharide K (KRESTIN): review of development and future perspectives. Surg Today 42: 8–28. 22139128 10.1007/s00595-011-0075-7 PMC3253283

[14] Geethangili M, Tzeng YM (2011) Review of pharmacological effects of *Antrodia camphorata* and its bioactive compounds. Evid Based Complement Alternat Med doi:10.1093/ecam/nep108 10.1093/ecam/nep108 PMC3095428 19687189

[15] Chen CJ, Su CH, Lan MH (2011) Study on solid cultivation and bioactivity of *Antrodia camphorate* . Fungal Sci 16: 65–72.

[16] Lee SB, Jeon HW, Lee YW, Lee YML, Song KW, et al (2003) Bio-artificial skin composed of gelatin and (1,3), (1,6)-glucan. Biomaterials 24: 2503–2511. 12695077 10.1016/s0142-9612(03)00003-6

[17] Moradali MF, Mostafavi H, Ghods S, Hedjaroude GA (2007) Immunomodulating and anticancer agents in the realm of macromycetes fungi (macrofungi). Int Immunopharmacol (7) 701–724. 10.1016/j.intimp.2007.01.008 17466905

[18] Chiu CH, Peng CC, Ker YB, Chen CC, Lee A, et al (2014) Physicochemical characteristics and anti-inflammatory activities of a novel glycoprotein, antrodan, isolated from *Antrodia cinnamomea* mycelia. Molecules 19: 22–40. 10.3390/molecules19010022 PMC6271056 24451244

[19] Carbonero ER, Sassaki GL, Gorin PAG, Iacomini M (2002) A (1→6)-linked β-mannopyrananan, pseudonigeran, and a (1→4)-linked β-xylan, isolated from the lichenised basidiomycete *Dictyonema glabratum* . FEMS Microbiol Lett 206: 175–178. 11814659 10.1111/j.1574-6968.2002.tb11005.x

[20] Wang CL, Pi CC, Kuo CW, Zhuang YJ, Khoo KH, et al (2011) Polysaccharides purified from the submerged culture of *Ganoderma formosanum* stimulate macrophage activation and protect mice against *Listeria monocytogenes* infection. Biotechnol Lett 33: 2271–2278. 21744272 10.1007/s10529-011-0697-2

[21] Miranda KM, Espey MG, Wink DA (2001) A rapid, simple spectrophotometric method for simultaneous detection of nitrate and nitrite. Nitric Oxide 1: 62–71. 10.1006/niox.2000.0319 11178938

[22] Bradford MM (1976) A rapid and sensitive method for the quantitation of micro quantities of protein utilizing the principle of protein-dye binding. Anal Chem 72: 248–254. 10.1016/0003-2697(76)90527-3 942051

[23] Marklund S, Marklund G (1974) Involvement of the superoxide anion radical in the autoxidation of pyrogallol and a convenient assay for superoxide dismutase. Eur J Biochem 47: 469–474. 4215654 10.1111/j.1432-1033.1974.tb03714.x

[24] Aebi H (1984) Catalase in vitro. Methods Enzymol 105: 121–126. 6727660 10.1016/s0076-6879(84)05016-3

[25] Lawrence RA, Burk RF (1976) Glutathione peroxidase activity in selenium- deficient rat liver. Biochem Biophys Res Commun 71: 952–958. 971321 10.1016/0006-291x(76)90747-6

[26] Buege JA, Aust SD (1978) Microsomal lipid peroxidation. Methods Enzymol 52: 302–310. 672633 10.1016/s0076-6879(78)52032-6

[27] Chen H, Zhang M, Xie B (2004) Quantification of uronic acids in tea polysaccharide conjugates and their antioxidant properties. J Agric Food Chem 52: 3333–3336. 15161193 10.1021/jf0349679

[28] Lindequist U, Niedermeyer TH, Jülich WD (2005) The pharmacological potential of mushrooms. Evid Based Complement Alternat Med 2: 285–299. 16136207 10.1093/ecam/neh107 PMC1193547

[29] Mizuno T (1999) Bioactive substances in *Hericium erinaceus* (Bull: Fr.) Pers. (Yamabushitake), and its medicinal utilization. Int. J. Med. Mushrooms 1: 105–119.

[30] Liu F, Ooi VEC, Chang ST (1997) Free radical scavenging activities of mushroom polysaccharide extracts. Life Sci 60: 763–771. 9064481 10.1016/s0024-3205(97)00004-0

[31] Chen Y, Xie MY, Nie SP, Li C, Wang YX (2008) Purification, composition analysis and antioxidant activity of polysaccharide from the fruiting bodies of *Ganoderma atrum* . Food Chem 107: 231–241.

[32] Akira S, Taga T, Kishimoto T (1993) Interleukin-6 in biology and medicine. Adv Immunol 54: 1–78. 8379461 10.1016/s0065-2776(08)60532-5

[33] Gao B (2012) Hepatoprotective and anti-inflammatory cytokines in alcoholic liver disease. J Gastroenterol Hepatol 27S2: 89–93. 10.1111/j.1440-1746.2011.07003.x PMC3281557 22320924

[34] Damas P, Ledoux D, Nys M, Vrindts Y, De Groote D, et al (1992) Cytokine serum levels during severe sepsis in human: IL-6 as a marker of severity. Ann Surg 215: 362–365. 10.1097/00000658-199204000-00009 PMC1242452 1558416

[35] Neveu WA, Bernardo E, Allard JL, Nagaleekar V, Wargo MJ, et al. (2011) Fungal allergen β-glucans trigger p38 mitogen-activated protein kinase–mediated IL-6 translation in lung epithelial cells. Am J Respir Cell Mol Biol 45: 1133–1141. 10.1165/rcmb.2011-0054OC PMC3262672 21642586

[36] Mohamadin AM, Elberry AA, Elkablawy MA, Gawad HS, Al-Abbasi FA (2011) Montelukast, a leukotriene receptor antagonist abrogates lipopolysaccharide-induced toxicity and oxidative stress in rat liver. Pathophysiology 18: 235–242. 21419608 10.1016/j.pathophys.2011.02.003

[37] Yokoyama H, Mizukami T, Kamegaya Y, Fukuda M, Okamura Y, et al (1998) Formation of superoxide anion in the hepatic sinusoid after lipopolysaccharide challenge. Alcohol Clin Exp Res 22(3S): 133S–136S. 9622390 10.1111/acer.1998.22.s3_part1.133s

[38] Sebai H, Sani M, Yacoubi MT, Aouani E, Ghanem-Boughanmi N, et al (2010) Resveratrol, a red wine polyphenol, attenuates lipopolysaccharide-induced oxidative stress in rat liver. Ecotoxicol Environ Saf 73: 1078–1083. 20089305 10.1016/j.ecoenv.2009.12.031

[39] Boveris A, Alvarez S, Navarro A (2002) The role of mitochondrial nitric oxide synthase in inflammation and septic shock. Free Radic Biol Med 33: 1186–1193. 12398926 10.1016/s0891-5849(02)01009-2

[40] Yazar E, Er A, Uney K, Bulbul A, Avci GE, et al (2010) Effects of drugs used in endotoxic shock on oxidative stress and organ damage markers. Free Radic Res 44: 397–402. 20102316 10.3109/10715760903513025

[41] Green SJ, Scheller LF, Marletta MA, Seguin MC, Klotz FW, et al (1994) Nitric oxide: cytokine-regulation of nitric oxide in host resistance to intracellular pathogens. Immunol Lett 43: 87–94. 7537721 10.1016/0165-2478(94)00158-8

[42] Mungrue IN, Husain M, Stewart DJ (2002) The role of NOS in heart failure: lessons from murine genetic models. Heart Fail Rev 7 407–422: 39. 10.1023/a:1020762401408 12379825

[43] Fujimiya Y, Suzuki Y, Oshiman K, Kobori H, Moriguchi K, et al. (1998) Selective tumoricidal effect of soluble proteoglucan extracted from the Basidiomycete, *Agaricus blazei* Murill, mediated via natural killer cell activation and apoptosis. Cancer Immunol Immunother 46: 147–59 1998. 10.1007/s002620050473 PMC11037308 9625538

[44] Yoshimura M, Nishikawa A, Ihara Y, Taniguchi S, Taniguchi T (1995) Suppression of lung metastasis of B16 mouse melanoma by N-acetylglucosaminyltransferase III gene transfection. Proc Natl Acad Sci U S A 92: 8754–8758. 7568011 10.1073/pnas.92.19.8754 PMC41045

[45] Fuster MM, Esko JD (2005) The sweet and sour of cancer: glycans as novel therapeutic targets. Nat Rev Cancer 5: 526–542. 16069816 10.1038/nrc1649

[46] Dennis JW, Pawling J, Cheung P, Parttridge E, Demetriou M (2002) UDP-N-acetylglucosamine: alpha-6-D-mannoside beta1,6 N-acetylglucosaminyl transferase V (Mgat5) deficient mice. Biochim Biophys Acta 1573: 414–422. 10.1016/s0304-4165(02)00411-7 12417426

[47] Tsuboi S, Hatakeyama S, Ohyama C, Fukuda M (2012) Two opposing roles of *O*-glycans in tumor metastasis. Trends Mol Med 18: 224–232. 10.1016/j.molmed.2012.02.001 PMC3356160 22425488

